# The Impact of Primary Care Physician Capacity on Preventable Hospitalizations: Identifying Bright Spots in the Appalachian & Mississippi Delta Regions

**DOI:** 10.13023/jah.0603.06

**Published:** 2024-10-01

**Authors:** Michael Topmiller, Peter J. Mallow, Hoon Byun, Mark Carrozza, Yalda Jabbarpour

**Affiliations:** American Academy of Family Physicians, The Robert Graham Center for Policy Studies in Family Medicine; Xavier University, Department of Health Services Administration; American Academy of Family Physicians, The Robert Graham Center for Policy Studies in Family Medicine; American Academy of Family Physicians, The Robert Graham Center for Policy Studies in Family Medicine; American Academy of Family Physicians, The Robert Graham Center for Policy Studies

**Keywords:** Appalachia, bright spots, Mississippi Delta, preventable hospitalization, primary care

## Abstract

**Introduction:**

Several studies have documented that higher rates of primary care physicians are associated with lower rates of preventable hospitalizations. Counties with higher rates of preventable hospitalizations are found in the Appalachian and Mississippi (MS) Delta Regions.

**Purpose:**

(1) To determine if the association of primary care capacity with preventable hospitalizations is different in the Appalachian and MS Delta regions compared to the rest of the U.S., and (2) to explore primary care capacity in counties with lower-than-expected preventable hospitalization rates.

**Methods:**

This study modeled preventable hospitalizations with primary care physicians (PCP) per 100,000 (PCP capacity) while controlling for several factors. A spatial regime variable was also included, which modeled Appalachian and MS Delta regions separately. Next, PCP capacity was removed from the model and a geospatial residual analysis was performed to identify geographic clusters of counties with lower-than-expected rates of preventable hospitalizations (bright spots). PCP capacity in bright spots was then compared to that in counties with higher-than-expected rates (cold spots).

**Results:**

Higher PCP capacity was significantly associated with lower rates of preventable hospitalizations in the rest of U.S. model, though was not significant for the Appalachian or MS Delta models. The residual analysis showed that compared to counties with higher-than-expected rates (cold spots), counties with lower-than-expected rates (bright spots) had significantly higher PCP capacity, though not in the MS Delta region.

**Implications:**

Consistent with previous literature, it was found that the factors associated with preventable hospitalizations vary by region, though the results are mixed when looking at the Appalachian and MS Delta regions separately. Future research should explore characteristics of bright spots within the Appalachian and MS Delta regions.

## INTRODUCTION

The rate of preventable hospitalizations is often measured by hospitalizations for ambulatory care sensitive conditions (ACSCs), which are conditions that can be effectively treated with outpatient care. Early intervention can prevent complications in these conditions. ACSCs include bacterial pneumonia, hypertension, diabetes, and chronic obstructive pulmonary disease.^1^–^2^ Preventable hospitalizations have been used as a proxy for the quality of and access to primary care^2^ and have also been associated with higher health care costs and poor health outcomes.^3^–^4^ Several studies have documented that multiple factors contribute to the occurrence of preventable hospitalizations, including poverty, lack of health insurance, language barriers, shortage of primary care physicians, and other geographic barriers.^5^–^7^

There are clear geographic patterns for preventable hospitalization, with several studies finding higher rates of preventable hospitalizations in the Appalachian and Mississippi (MS) Delta Regions.^8^–^10^ Weiss and Jiang explored sub-state variation in potentially preventable inpatient stays and found significant geographic variation across states. They identified several geographic clusters of areas in the top quartile for preventable hospital stays, referred to as hot spot regions, including the MS Delta and Appalachian Regions.^8^ Utilizing Medicare data focused on preventable hospitalizations for hypertension, another study used the Local Moran’s I to identify geographic clusters of counties with high rates of preventable hospitalization, with many of them located in Central Appalachia and the MS Delta.^9^ Also using Medicare data, Lin et al. utilized geographic weighted regression to explore the association between primary care access and preventable hospitalizations, finding preventable hospitalization hot spots primarily in the MS Delta and Appalachian Regions.^10^

In addition to research finding higher rates of preventable hospitalization in the Appalachian and MS Delta Regions, other studies have found that factors influencing preventable hospitalizations may not be consistent across geographic space.^10^–^11^ As mentioned above, Lin et al. utilized a geographically weighted regression and discovered geographic variation in the association of primary care access on preventable hospitalizations. Other research found that black-white disparities in preventable hospitalizations differed based by Medicare plan type and geography.^10^–^11^

A few methodological approaches exist for testing how factor coefficients for independent variables vary by geographic region, including spatial regime models.^12^ Spatial regime models create spatial subsets within the model and allow coefficients to vary for each spatial subset.^12^ There are few examples of spatial regime models used in healthcare research.^13^–^15^ Grekousis found factors associated with county-level COVID-19 death rates varied by metropolitan status, political affiliation, and policy.^13^ Myers 2014, 2016 utilized spatial regime models to examine factors associated with diabetes and obesity, finding that factors did vary by geographic region.^14^–^15^

While previous research has explored the geographic variation of preventable hospitalizations and the impact of primary care physician (PCP) rates per population, no studies have looked explicitly at how PCP rates and other factors associated with preventable hospitalization may differ within high-need regions such as Appalachia. Thus, the first aim of this research is to determine if the association of PCP rates per 100,000 with preventable hospitalizations is different in the Appalachian and MS Delta Regions, compared to the rest of the U.S. The second aim of this research is to identify counties with lower-than-expected preventable hospitalization rates (bright spots) and compare their characteristics with counties with higher-than-expected preventable hospitalization rates (cold spots). This positive deviance approach builds on previous bright spot research that aims to identify and characterize high-performing geographic areas.^16^–^17^

## METHODS

### Data and Measures

This cross-sectional study used publicly available, county-level data. The primary outcome measure was the rate of preventable hospitalizations, which is defined as hospital stays for ACSCs per 100,000 Medicare enrollees – these data came from the County Health Rankings (2023). Independent variables were selected based on previous literature examining factors associated with preventable hospitalizations. These four factors included (1) the percentage of black Medicare beneficiaries; (2) hospital beds per 100,000 and hierarchical condition category (HCC) risk scores (from the Centers for Medicare and Medicaid geographic variation public use files, 2021); (3) primary care physicians (PCP) per 100,000 and the percentage of population in rural areas (also from the County Health Rankings); and (4) the social deprivation index (SDI) [from the Robert Graham Center (2017–2021)].^18^–^20^ HCC risk scores, which are calculated using Medicare beneficiaries demographic information and medical conditions, can be used as a proxy for overall health – meaning higher HCC risk scores indicate less healthy Medicare populations.^11^ The social deprivation index (SDI), which is a composite measure of several socio-economic variables including poverty, education, employment, and housing, has been associated with less access to primary care and worse health outcomes.^20^

### The Mississippi Delta & Appalachian Regions

The Mississippi (MS) Delta and Appalachian Regions are home to more than 35 million people and 672 counties.^21^–^22^ The MS Delta Region includes 252 counties in eight states and shares ten counties with the Appalachian Region in Alabama and Mississippi.^22^–^23^ Using geographic, racial/ethnic, and socioeconomic characteristics as a guide, seven Mississippi counties were assigned to the MS Delta Region, and three Alabama counties were assigned to the Appalachian Region.

### Analysis (Phase I – Building the Regression Model)

Counties with missing data were removed, including 32 counties in the MS Delta Region and 48 counties in Appalachia. Ordinary least squares (OLS) was used to model preventable hospitalizations with PCPs per 100,000; HCC risk scores; SDI; rurality; hospital beds per 100,000; and the percentage of black Medicare beneficiaries as independent variables. All variables were standardized to account for heteroskedasticity.

A spatial regime variable was also included, which created spatial subsets for counties in the Appalachian and MS Delta Regions and modeled them separately from the rest of U.S. counties, resulting in three separate regression models. A Chow test was then used to determine if coefficients are significantly different across the three models.^12^

### Analysis (Phase II - Geospatial Residual Analysis)

The second phase of the analysis involved removing primary care capacity from the OLS model and performing a residual analysis of counties with bright spots and cold spots rates of preventable hospitalizations. Bright spots and cold spots were determined using the Local Moran’s I to identify statistically significant clusters; the Local Moran’s I is a local indicator of spatial autocorrelation that identifies statistically significant geographic clusters (for example, high rates of preventable hospitalization) by comparing observed patterns against randomized patterns.^12^ Finally, primary care physician capacity and other characteristics of bright spot and cold spot counties were compared.

## RESULTS

Table 1 displays the descriptive results for Appalachia, MS Delta, and rest of U.S. geographic counties for the study’s outcome measure and independent variables. As expected, preventable hospitalizations were significantly higher for the MS Delta and Appalachian Regions compared to the rest of the U.S. The Appalachian and MS Delta Regions also were more rural and had significantly higher rates of morbidity (HCC risk scores) and social deprivation – though the MS Delta Region had significantly higher rates than Appalachia for both measures. The MS Delta Region also had significantly higher rates of hospital beds per 100,000 and percentages of black beneficiaries, while having significantly lower rates of PCPs per 100,000.

### Model Results

Table 2 displays the results from the study’s OLS spatial regime model that included PCPs per 100,000 as an independent variable. The spatial regime modeled the three regions separately, resulting in a separate r^2^ and coefficients for each. The rest of U.S. model performed better (r^2^ = .32) compared to the MS Delta (r^2^ = .17) and Appalachian Region (r^2^ = .30) models. PCPs per 100,000 had a negative, statistically significant impact on preventable hospitalizations rates in the rest of U.S. model but was not statistically significant for the Appalachian Region or MS Delta models. All independent variables were statistically significant in the rest of U.S. model, while only three variables were significant in the MS Delta and Appalachian Region models (SDI, hospital beds, HCC risk scores). Table 2 also reveals the Chow test results – *p*-values of less than .05 show that coefficients varied significantly across the models for all independent variables, except SDI and PCP rates.

### Identifying Bright Spots

**Figure 1:**
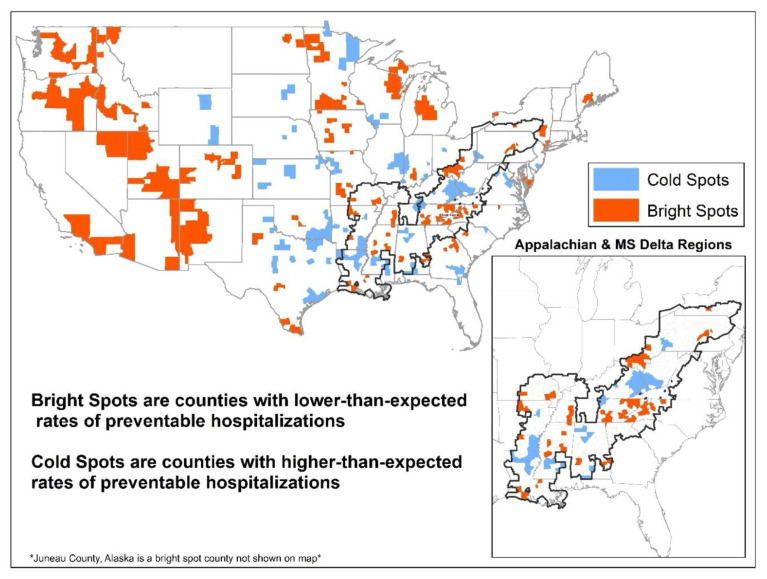
Bright Spots and Cold Spots

Figure 1 displays the results from the residual analysis. Counties with lower-than-expected preventable hospitalization rates (bright spots, n=251) were located throughout the U.S., with clusters of bright spot counties in the Western U.S., Michigan, Wisconsin, Minnesota, and in the Appalachian Region. A disproportionate share of bright spots and cold spots were in the Appalachian and MS Delta Regions – 27% of bright spots and 34% of cold spots are located in these regions. Figure 1 also shows clusters of bright spot counties located close to clusters of cold spot counties within the MS Delta and Appalachian Regions.

Table 3 shows the median characteristics of bright spot and cold spot counties by region (rest of U.S., Appalachia, MS Delta). PCP capacity was higher in bright spot counties in rest of U.S. and in Appalachia, though not in MS Delta region. The same pattern holds for rurality, as bright spots were also less rural than cold spots in the rest of U.S. and Appalachian Regions. Bright spots in all regions had lower rates of morbidity (HCC risk scores), social deprivation, and hospital bed rates compared to cold spots within the same region.

## IMPLICATIONS

Consistent with previous literature, this research finds that primary care physician rates per 100,000 is associated with lower rates of preventable hospitalizations, though this varies by geographic region. PCP rates have a negative, statistically significant association in the rest of the U.S. model but is not statistically significant for the Appalachian and MS Delta Region models. Further, the Chow test determines that the coefficient for PCP rates do not vary across the three models, suggesting that while having more PCPs per 100,000 may be important for improving rates of preventable hospitalizations in the Appalachian and MS Delta Regions, other social, economic, and health factors are more important. Moreover, the only factors significantly associated with preventable hospitalizations across all three models are SDI, which is a composite social determinant of health measure, HCC risk scores (morbidity), and hospital bed rates, which higher rates have been found to be associated with increased rates in hospitalization.^24^

Looking at bright spot counties within the three regions provides further insights into the challenges associated with reducing rates of preventable hospitalizations in the Appalachian and MS Delta Regions. Cold spots have higher rates of social deprivation and HCC scores for all regions – indicating higher rates of morbidity and challenges related to social determinants for these areas compared to bright spots. Bright spots in the rest of the U.S. and Appalachian Regions have higher rates of PCPs per 100,000 and are less rural compared to cold spots in their respective regions. The same is not true for the MS Delta Region, where bright spots are more rural, have lower rates of PCPs and fewer hospital beds, suggesting that these areas may utilize less health care overall, though further study is needed. Finally, the higher rates of black Medicare beneficiaries in MS Delta Region cold spots (compared to bright spots) suggest that race is another important factor, which is consistent with the literature.^25^

This research points to the need for mixed methods studies in high-need regions that can explain the root causes of preventable hospitalizations beyond what is currently measured and how this can help tailor interventions to improve public health. Our previous bright spot work provides a potential guide for identifying peer counties (bright spots and cold spots) within these high-need regions, as well as engaging in qualitative research to better understand how cold spots can learn from bright spots.^17^ Potential examples can be found in Appalachian Tennessee, where a cluster of cold spot counties are located in the north-central part of the state while many bright spot counties are clustered in south-central Tennessee.

This research has a few limitations related to small number of observations and missing data – about 14% of counties were removed due to missing data, including many in the Appalachian and MS Delta Regions. These missing data counties are more rural, have smaller populations, and likely require their own in-depth studies to determine factors associated with preventable hospitalizations. Another limitation related to the data is the smaller number of observations in the Appalachian and MS Delta Regions compared to the U.S. and the large degree of variation in the rest of the U.S. model – which included counties from across the U.S. Better understanding of preventable hospitalizations will require future research to focus on specific areas within U.S. regions and the use of mixed methods approaches.

**SUMMARY BOX**

**What is already known about this topic?**

The Appalachian and MS Delta Regions have higher rates of preventable hospitalizations compared to other parts of the U.S.

**What is added by this report?**

This research explored how primary care capacity and other factors associated with preventable hospitalization vary by geographic region and identified preventable hospitalization bright spots (counties with lower-than-expected rates).

**What are the implications for future research?**

Future research utilizing qualitative approaches can investigate unique characteristics of bright spot counties that can be applied to cold spot counties.

## Figures and Tables

**Table 1:** Descriptive Statistics by Region

	Appalachian	MS Delta	Rest of U.S.
# of counties	372	230	2,085
**Outcome Measure**			
Preventable Hospitalizations per 100,000 Medicare enrollees	3,596	3,844	2,836
**Independent Variables**			
% Rural	67.0	64.5	52.6
% Black (Medicare Beneficiaries)	2.6	10.7	4.2
Hierarchical Condition Category (HCC)	1.00	1.04	0.96
Social Deprivation (SDI)	57.4	73.1	43.7
Primary Care Physician Rate per 100,000	49.3	40.5	57.4
Hospital Beds per 100,000	30,747	46,319	34,922

**Table 2:** Model Results (Coefficients, *p*-value)

	Appalachian Model	MS Delta Model	Rest of U.S. Model	Chow Test *p*-values
**# of Counties**	372	230	2,085	
Adjusted R-Squared	.2951	.1705	.3245	.000
% Rural	−0.143 (.068)	−0.056 (ns)	0.074 (.000)	.01
% Black (Medicare Beneficiaries)	−0.171 (.059)	0.010 (ns)	0.126 (.000)	.000
Hierarchical Condition Category (HCC)	5.530 (.000)	1.911 (.055)	3.970 (.000)	.02
Social Deprivation (SDI)	0.235 (.008)	0.241 (.031)	0.081 (.000)	.09
Primary Care Physician Rate per 100,000	−0.056 (ns)	0.014 (ns)	−0.082 (.000)	.57
Hospital Beds per 100,000	0.662 (.000)	0.477 (.000)	0.141 (.000)	.000

**Table 3:** Median Bright Spot & Cold Spot Characteristics (With Focus on Appalachian/MS Delta)

	Appalachian Bright Spots	Appalachian Cold Spots	MS Delta Bright Spots	MS Delta Cold Spots	Rest of US Bright Spots	Rest of US Cold Spots
**# counties**	48	43	20	24	183	127
**Outcome Measure**						
Preventable Hospitalizations per 100,000 Medicare enrollees	2,730	5,243	3,215	4,900	1,971	3,886
**Independent Variables**						
% Rural	70.6	81.4	67.3	65.3	48.5	61.3
% Black (Medicare Beneficiaries)	0.7	0.0	2.0	12.6	0.3	1.1
Hierarchical Condition Category (HCC)	1.00	1.01	1.03	1.06	0.95	0.99
Social Deprivation (SDI)	63.5	74.0	72.0	80.0	42.0	54.0
Hospital Beds per 100,000 PCP Capacity	23,180	44,611	27,675	52,472	11,837	29,218
Primary Care Physician Rate per 100,000	44.3	33.9	28.2	41.7	53.5	40.1
